# Physiological Adaptation Buffers Personality‐Linked Psychopathology Under High‐Altitude Hypoxia

**DOI:** 10.1002/brb3.71591

**Published:** 2026-07-09

**Authors:** Yuan Li, Niannian Wang, Zhibin Geng, Wenjie Liu, Hailin Ma, Hao Li

**Affiliations:** ^1^ Xizang Autonomous Region Key Laboratory For High Altitude Brain Science and Environmental Acclimatization Xizang University Lhasa China; ^2^ China Railway No.10 Engineering Group Co., Ltd. Jinan China

**Keywords:** hematologic compensation, high‐altitude hypoxia, interaction effect, personality, psychological health, psychoticism

## Abstract

**Purpose:**

High‐altitude hypoxia imposes persistent physiological stress that may influence psychological health. While personality traits such as neuroticism are known predictors of psychological symptom burden, little is known about whether physiological adaptation—specifically hematologic compensation—is associated with personality‐related differences in psychological symptoms under chronically hypoxic conditions.

**Methods:**

Healthy adults residing at high altitude completed the Chinese version of the Big Five Inventory‐2 and provided blood data for RBC, HGB, and HCT. *k*‐Means clustering identified two personality clusters (high vs. low neuroticism) and three hematologic compensation clusters (low, medium, and high). Psychological symptoms across nine domains were assessed using the Symptom Checklist‐90 (SCL‐90), a 90‐item self‐report inventory designed to assess a broad range of psychological symptoms.

**Finding:**

A 2 (personality) × 3 (hematologic compensation) multivariate general linear model revealed no significant interactions on most SCL‐90 dimensions after controlling for gender, age, and hypoxic exposure time, except for a significant interaction on the SCL‐90 psychoticism dimension (*F*(2, 120) = 4.026, *p* = 0.02, $\eta_{p}^{2}$ = 0.063). Simple‐effect analysis showed that in the high hematologic compensation group, individuals in the high‐neuroticism cluster reported lower SCL‐90 psychoticism scores than those in the low‐neuroticism cluster.

**Conclusion:**

These findings provide preliminary evidence for a domain‐specific interaction between personality profiles and hematologic compensation on the SCL‐90 psychoticism dimension under chronic high‐altitude hypoxia. Importantly, this dimension reflects self‐reported subclinical psychoticism‐related tendencies rather than clinical psychosis. Because this interaction was not observed across most SCL‐90 dimensions, the results should be interpreted cautiously and require replication.

## Graphical abstract

Among 129 healthy long‐term residents living at high altitude in Lhasa, Big Five personality traits and hematologic indices (RBC, HGB, and HCT) were clustered into 2 personality profiles and 3 hematologic compensation levels. A 2 × 3 multivariate general linear model showed a specific interaction on SCL‐90 psychoticism: within the high compensation group, the high‐neuroticism profile displayed lower psychoticism than the low‐neuroticism profile. These findings suggest that stronger hematologic adaptation may conditionally buffer personality‐related psychological vulnerability during chronic high‐altitude hypoxia.

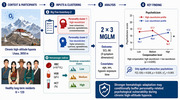

## 1 Introduction

Prolonged exposure to hypobaric hypoxia in high‐altitude environments poses significant physiological challenges and has increasingly been linked to adverse psychological effects. Hypoxia at high altitudes not only disrupts sleep, attention, and executive function but has also been associated with anxiety, depression (DE), and other self‐reported psychological symptom dimensions (Y. Li, Wang, et al. 2025; Hösl et al. 2025). Prior studies have shown that elevation‐related hypoxia may impair emotional regulation and cognitive processing, especially in individuals lacking sufficient physiological adaptation (Xu et al. 2025). Therefore, high‐altitude exposure serves not only as a physiological stressor but also as a unique context to explore mechanisms of psychological adaptation.

Personality traits have long been recognized as fundamental psychological factors influencing individual differences in mental health. According to the Five‐Factor Model, neuroticism reflects an individual's susceptibility to negative emotions and is among the strongest predictors of psychological distress. A wealth of research has shown that higher neuroticism is closely associated with higher levels of anxiety, DE, hostility (HO), and other psychological symptom tendencies (Widiger and Oltmanns 2017; Lahey 2009). In contrast, traits such as extraversion, conscientiousness, and agreeableness are often linked to greater psychological adaptation (Ormel et al. 2013). Under the chronic stress of high‐altitude hypoxia, individuals high in neuroticism may exhibit maladaptive appraisals and emotional responses, which may increase their vulnerability to psychological distress or symptom expression. Thus, investigating personality profiles provides a crucial framework for understanding psychological adaptation in extreme environments such as high‐altitude regions.

Notably, physiological adaptation to high‐altitude hypoxia—particularly hematologic compensation—may play a vital role in modulating psychological health. Key hematologic markers such as hemoglobin (HGB), red blood cell count (RBC), and hematocrit (HCT) are widely recognized as indicators of oxygen transport efficiency under hypoxic conditions. Recent findings show that individuals with higher levels of hematologic adaptation tend to exhibit more stable cognitive performance and lower psychological stress in hypoxic environments (Gong et al. 2025). Moreover, in simulated acute high‐altitude exposure, circulating erythropoietin (EPO) levels were found to be closely associated with fatigue and executive functioning outcomes (Stalmans et al. 2025). Although these outcomes are not direct measures of mental health, they are functionally relevant to psychological adaptation under hypoxic stress. Fatigue may contribute to subjective distress and reduced coping capacity, whereas executive functioning supports cognitive control, emotion regulation, and adaptive responses to environmental demands (Heinrich et al. 2019; Ochsner and Gross 2008). These findings suggest that physiological adaptation to high‐altitude hypoxia may be relevant not only to oxygen transport and physical functioning but also to broader psychological functioning in high‐altitude environments. Yet, few studies have investigated whether such physiological factors can moderate the relationship between personality traits and psychological outcomes. The present study addresses this gap by exploring the interactive effects of personality profiles and hematologic compensation on multiple dimensions of psychological symptoms.

In our previous report using the same high‐altitude cohort, we identified distinct hematologic compensation profiles and demonstrated that hematologic adaptation, together with brain structural variation, was associated with reduced depressive symptoms under chronic hypoxia (Li et al. 2025). That study provided initial evidence that hematologic adaptation may be linked to psychological symptoms in relation to brain structural characteristics. However, the previous analysis focused on depressive symptoms and brain structural variation, and did not examine whether hematologic compensation conditions the relationship between stable personality dispositions and broader psychological symptom dimensions. The present study, therefore, extends our previous work by integrating Big Five personality profiles and multi‐domain Symptom Checklist‐90 (SCL‐90) symptoms, and by testing personality cluster × hematologic compensation interactions.

In summary, high‐altitude environments offer a unique natural context for exploring the complex interplay between personality traits, physiological adaptation, and psychological well‐being. In this study, we applied cluster analysis to both the Five‐Factor Model of personality and hematologic indicators (HGB, RBC, and HCT), identifying distinct personality profiles and levels of hematologic compensation. Utilizing a 2 (personality cluster) × 3 (hematologic compensation cluster) multivariate general linear model (MGLM), we examined the main and interactive effects of these cluster types on nine dimensions of psychological symptoms, as measured by the SCL‐90, a 90‐item self‐report inventory assessing a broad spectrum of psychological symptoms. Based on the literature reviewed above and our previous findings in the same high‐altitude cohort, we proposed the following hypotheses: H1, personality profiles would be associated with psychological symptom scores, with the high‐neuroticism profile showing higher symptom scores, particularly in negative‐affect‐related domains; H2, hematologic compensation level would be associated with psychological symptom scores under chronic high‐altitude hypoxia; and H3, personality profiles and hematologic compensation would interact in relation to psychological symptoms, such that the association between personality profile and symptom scores would differ across hematologic compensation levels. Because the present study examined multiple SCL‐90 dimensions and prior evidence did not allow precise predictions for each subscale, the specific symptom domains showing interaction effects were treated as exploratory and interpreted cautiously.

## 2 Methods

### 2.1 Participants

A total of 129 Han Chinese adults currently living, working, or studying in Lhasa, Xizang, were recruited from a larger ongoing project on long‐term high‐altitude adaptation. Recruitment was conducted in the Lhasa area through local institutional and community channels. Eligible participants were adults who were born and raised at low altitude and had migrated to Lhasa in adulthood for work or study. To be included in the present analysis, participants were required to have completed the Big Five Inventory‐2, the SCL‐90 assessment, and routine hematologic testing, including RBC, HGB concentration, and HCT, at the local hospital.

The sample included 67 females (mean age = 34.8 ± 3.7 years) and 62 males (mean age = 35.0 ± 3.4 years). Regarding educational attainment, 36% held a master's degree, 33% had a bachelor's degree, and 31% had less than a bachelor's degree. In terms of occupation, 61% were employed professionals, while 39% were self‐employed workers.

All participants had been long‐term residents of the Lhasa area, with a mean high‐altitude exposure duration of 10.7 ± 4.0 years, and resided in Xizang for an average of 10.1 ± 0.7 months per year. They were all born and raised at low altitudes and migrated to Lhasa, which has an average altitude of approximately 3650 m, for work or study in adulthood. The participant characteristics stratified by sex are summarized in Table 1.

**TABLE 1:** Participant characteristics stratified by sex.

Variable	Female (*n* = 67)	Male (*n* = 62)	Total (*N* = 129)
Age, years	34.8 ± 3.7	35.0 ± 3.4	34.9 ± 3.6
High‐altitude exposure duration, years	11.0 ± 4.4	10.4 ± 3.6	10.7 ± 4.0
Annual residence time in Xizang, months/year	10.1 ± 0.7	10.1 ± 0.6	10.1 ± 0.7
Education, *n* (%)			
Less than bachelor's degree	18 (45%)	22 (55%)	40 (31%)
Bachelor's degree	23 (53%)	20 (47%)	43 (33%)
Master's degree	25 (54%)	21 (46%)	46 (36%)
Occupation, *n* (%)			
Employed professionals	44 (56%)	35 (44%)	79 (61%)
Self‐employed workers	21 (42%)	29 (58%)	50 (39%)

All participants had normal or corrected‐to‐normal vision, no color blindness, and no history of psychiatric disorders, traumatic brain injury, hypertension, cardiovascular disease, or other major medical conditions. Written informed consent was obtained from all participants prior to the experiment. All participants volunteered to participate in this study and received monetary compensation upon completion of the blood test and questionnaire assessments.

Because participation was voluntary and required completion of both questionnaire assessments and hospital‐based hematologic testing, possible selection bias should be acknowledged. The sample may have overrepresented relatively healthy, stable, and motivated long‐term high‐altitude residents. In addition, the exclusion of individuals with psychiatric disorders or major medical conditions may limit the generalizability of the findings to broader high‐altitude populations or clinically vulnerable groups.

The present dataset was derived from a larger ongoing project on long‐term high‐altitude adaptation in Lhasa. Portions of this cohort, including routine hematologic indices (RBC, HGB, and HCT) and basic cohort characteristics, have been reported previously in our prior publication (Li et al. 2025), which focused on hematologic profiles, brain structure, and depressive symptoms. The current manuscript addresses distinct research questions by integrating Big Five personality profiles and broad SCL‐90 symptom dimensions and by testing personality cluster × hematologic compensation interactions. No analyses of personality profiles or personality‐by‐hematologic compensation interactions were reported in the previous study.

### 2.2 Behavioral Measures

The Big Five personality traits were measured using the Chinese version of the Big Five Inventory‐2 (Zhang et al. 2021). The inventory consists of 60 questions scored on a 5‐point Likert scale (1 = *strongly disagree* and 5 = *strongly agree*). The inventory has demonstrated good reliability and validity in previous studies (Bai et al. 2024; M. Li et al. 2024; Liu et al. 2024). In this study, its Cronbach's *α* was 0.81.

Psychological symptoms were assessed using the Chinese version of the SCL‐90 (Dang et al. 2021). This scale consists of 90 items and is divided into 10 factors, each reflecting a different aspect of psychological symptoms. Importantly, the psychoticism dimension of the SCL‐90 was treated as a self‐reported subclinical symptom dimension reflecting interpersonal alienation, social withdrawal, unusual perceptual experiences, and thought‐related discomfort, rather than as an indicator of clinical psychosis or psychotic disorder. The scale uses a 5‐point scoring system, where “1” represents “*none—no awareness of the symptom or problem*,” and “5” represents “*severe—the frequency and intensity of the symptom are both very severe*.” It has shown good reliability and validity in previous studies (Du et al. 2022; Xu et al. 2022). In this study, the Cronbach's *α* coefficient was 0.87.

### 2.3 Hematological Data Acquisition and Analysis

Participants underwent routine hematological testing at a local hospital in Lhasa, Xizang. Blood samples were analyzed by the hospital's clinical laboratory, which also provided standardized medical reports. The RBC, HGB, and HCT measures analyzed here overlap with those reported in our prior publication (Li et al. 2025), which focused on hematologic profiles in relation to brain structure and depressive symptoms. The present study differs by examining personality profiles and multi‐domain psychological symptoms (SCL‐90) and by testing personality‐by‐hematologic interactions.

### 2.4 Statistical Analysis

Two independent *k*‐means clustering procedures were conducted in the R environment. The first clustering procedure was performed to identify hematologic compensation profiles based on three hematologic indicators: RBC, HGB concentration, and HCT. Before clustering, all three variables were standardized using the scale() function to remove differences in measurement scales. The final number of clusters was determined by considering statistical criteria, interpretability, and parsimony. For the hematologic indicators, the elbow method based on within‐cluster sum of squares showed that the improvement in model fit became smaller after *k* = 3. Therefore, the three‐cluster solution was selected. Based on the relative levels of RBC, HGB, and HCT, Hematologic Cluster 1 was interpreted as the low hematologic compensation group, Hematologic Cluster 2 as the medium hematologic compensation group, and Hematologic Cluster 3 as the high hematologic compensation group.

The second clustering procedure was performed to identify personality profiles based on the five Big Five personality traits: neuroticism, extraversion, openness, agreeableness, and conscientiousness. These variables were also standardized before clustering. Candidate cluster solutions were evaluated using the elbow method and the silhouette coefficient, together with interpretability and parsimony. The two‐cluster solution was retained because it provided a clear and theoretically interpretable distinction between personality profiles. Personality Cluster 1 was characterized by relatively higher neuroticism and lower conscientiousness, agreeableness, openness, and extraversion and was therefore interpreted as the high‐neuroticism personality profile. Personality Cluster 2 was characterized by relatively lower neuroticism and higher scores on the other four Big Five traits and was therefore interpreted as the low‐neuroticism personality profile.

The two resulting clustering variables were then used as categorical factors in the main statistical model. Specifically, a 2 (personality cluster: high‐neuroticism vs. low‐neuroticism) × 3 (hematologic compensation cluster: low, medium, and high) MGLM was conducted in SPSS 26.0 to examine their main and interactive effects on the nine SCL‐90 symptom dimensions. Gender, age, and hypoxic exposure time were included as covariates. When a significant interaction was observed, simple‐effect analyses were conducted to characterize the pattern of group differences. In the present study, simple‐effect analyses were performed for the SCL‐90 psychoticism dimension because this was the only dimension showing a significant personality cluster × hematologic compensation cluster interaction.

## 3 Results

### 3.1 Hematologic Compensation Clusters Based on RBC, HGB, and HCT

**FIGURE 1:**
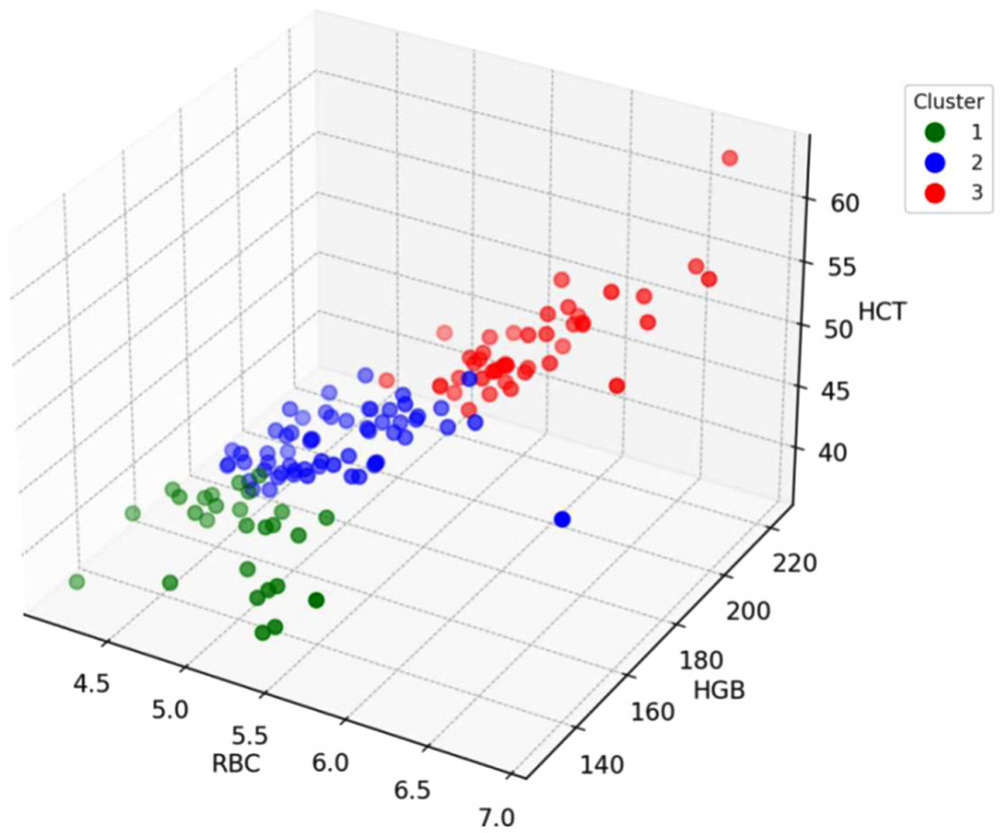
Three‐dimensional visualization of *k*‐means clustering results based on RBC, HGB, and HCT.

The descriptive statistics for the three hematologic compensation clusters are presented in Table 2 and illustrated in Figure 1.

**TABLE 2:** Descriptive statistics for hematologic compensation clusters (*N* = 129).

Clusters	*n*	RBC (M ± SD, ×10^12^/L)	HGB (M ± SD, g/L)	HCT (M ± SD, %)
1 (Low)	27	5.0 ± 0.4	141.4 ± 7.8	41.5 ± 1.8
2 (Medium)	59	5.3 ± 0.4	161.7 ± 6.7	46.2 ± 1.8
3 (High)	43	6.1 ± 0.3	186.7 ± 9.6	52.4 ± 2.9

### 3.2 ANOVA Results for Hematological Indices Across Clusters

**FIGURE 2:**
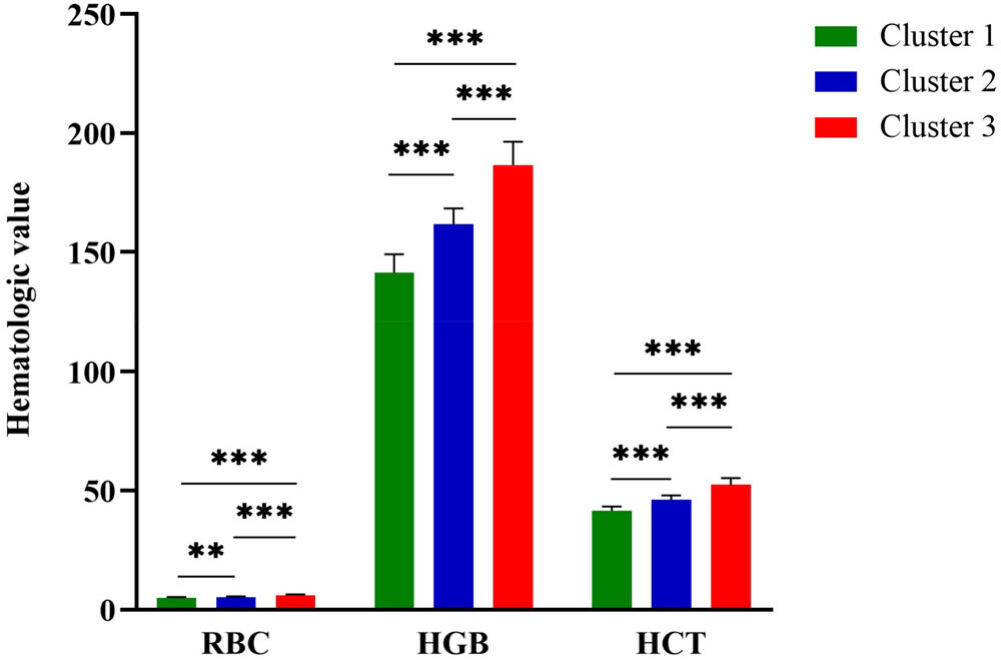
Differences in RBC, HGB, and HCT levels across the three hematologic compensation clusters. **p < 0.01, ***p < 0.001.

A one‐way ANOVA was conducted to examine differences in RBC, HGB, and HCT levels across the three hematologic compensation clusters. Significant cluster differences were observed for RBC (*F*(2, 126) = 97.63, *p* < 0.001, $\eta_{p}^{2}$ = 0.608), HGB (*F*(2, 126) = 278.84, *p* < 0.001, $\eta_{p}^{2}$ = 0.816), and HCT (*F*(2, 126) = 215.30, *p* < 0.001, $\eta_{p}^{2}$ = 0.774). Bonferroni post hoc tests confirmed a clear stepwise pattern, with Cluster 3 showing the highest values, Cluster 2 intermediate values, and Cluster 1 the lowest values for all three hematologic indicators. These results support the interpretation of the three clusters as high, medium, and low hematologic compensation groups, respectively. The results are illustrated in Figure 2.

### 3.3 Personality Clusters Based on Big Five Traits

**FIGURE 3:**
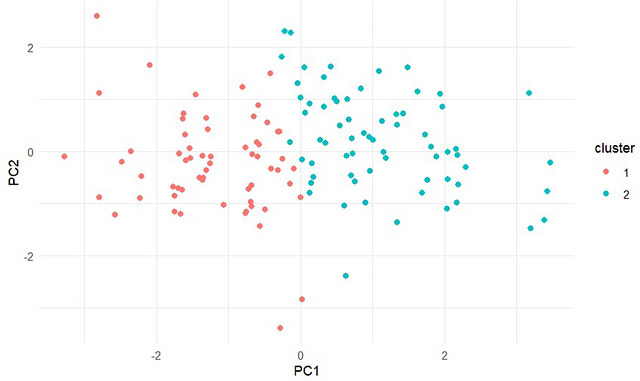
PCA‐based visualization of k‐means clustering results for the Big Five personality traits.

The descriptive statistics for the two clusters of personality traits are presented in Table 3 and illustrated in Figure 3.

**TABLE 3:** Descriptive statistics for personality clusters (M ± SD; *N* = 129).

Clusters	*n*	Neuroticism	Conscientiousness	Agreeableness	Openness	Extraversion
1	62	25.30 ± 6.00	30.07 ± 3.88	32.58 ± 4.84	28.23 ± 5.84	26.72 ± 5.18
2	67	21.09 ± 6.83	38.34 ± 4.12	39.69 ± 3.90	35.85 ± 5.79	30.98 ± 7.32

### 3.4 *t*‐Test Results for the Big Five Personality Traits Across Clusters

**FIGURE 4:**
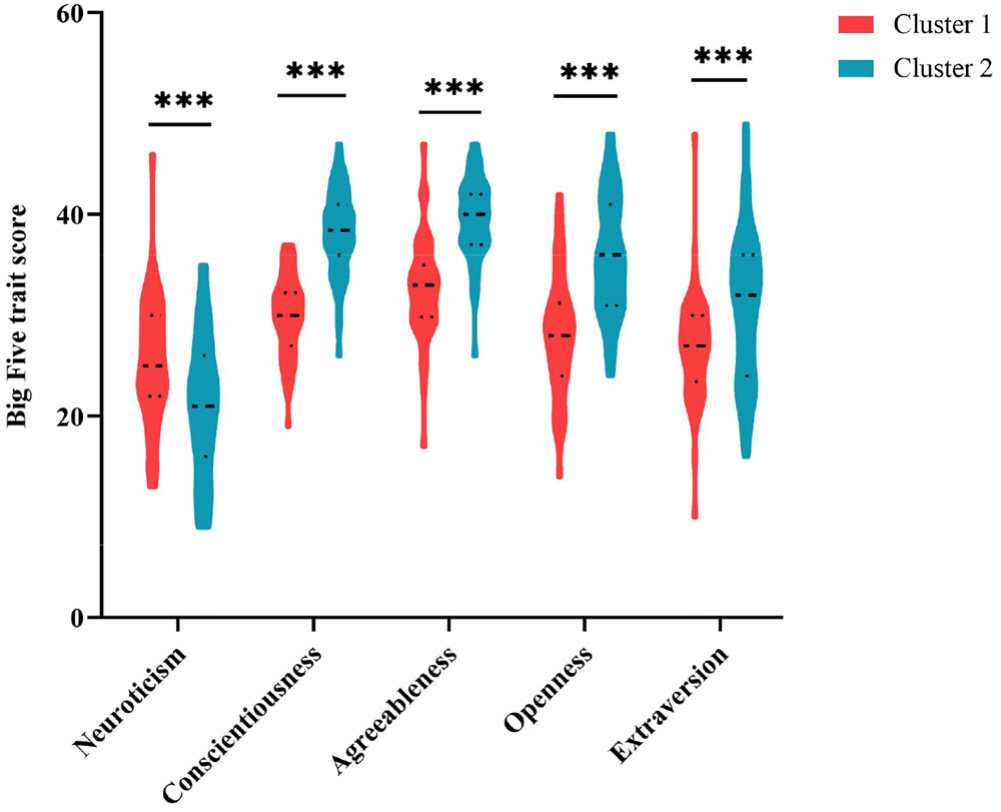
Differences in Big Five trait scores between the two personality clusters. ***p < 0.001.

Independent‐samples *t*‐tests confirmed that Personality Cluster 1 had significantly higher neuroticism scores and significantly lower conscientiousness, agreeableness, openness, and extraversion scores than Personality Cluster 2 (all *ps* < 0.001). Thus, Cluster 1 was interpreted as the high‐neuroticism profile and Cluster 2 as the low‐neuroticism profile. These results are illustrated in Figure 4.

### 3.5 Effects of Personality Cluster and Hematologic Compensation Cluster on SCL‐90 Dimensions

The results showed that there was only an interaction between personality trait clustering and blood compensation level on the SCL‐90 psychoticism dimension (*F*(2, 120) = 4.026, *p* = 0.02, $\eta_{p}^{2}$ = 0.063) after controlling for gender, age, and hypoxic exposure time.

In addition, personality cluster showed a significant main effect on DE (*F*(1, 120) = 8.239, *p* = 0.005, $\eta_{p}^{2}$ = 0.064), with higher DE scores in the high‐neuroticism cluster than in the low‐neuroticism cluster. Hematologic compensation cluster showed significant main effects on obsessive‐compulsive (OC) symptoms (*F*(2, 120) = 3.139, *p* = 0.047, $\eta_{p}^{2}$ = 0.050) and HO (*F*(2, 120) = 5.381, *p* = 0.006, $\eta_{p}^{2}$ = 0.082). Post hoc comparisons indicated that the high compensation group reported lower OC and HO scores than the medium compensation group. Full statistical results are presented in Table 4.

**TABLE 4:** MGLM results for personality cluster, hematologic compensation cluster, and their interaction on SCL‐90 dimensions.

Source	Dependent variable	Type III SS	*df*	Mean square	*F*	Sig. (*p*‐value)	Partial *η* ^2^
Personality traits clustering	Somatization (SO)	129.751	1	129.751	3.917	0.050	0.032
	Obsessive‐compulsive (OC)	0.058	1	0.058	0.003	0.959	< 0.001
	Interpersonal sensitivity (IS)	31.589	1	31.589	1.697	0.195	0.014
	Depression (DE)	252.685	1	252.685	8.239	0.005	0.064
	Anxiety (AN)	20.423	1	20.423	1.029	0.312	0.009
	Hostility (HO)	9.733	1	9.733	0.998	0.320	0.008
	Phobic anxiety (PA)	12.177	1	12.177	1.839	0.178	0.015
	Paranoid ideation (PI)	3.413	1	3.413	0.476	0.492	0.004
	Psychoticism (PS)	2.861	1	2.861	0.196	0.659	0.002
Blood compensatory levels	Somatization (SO)	47.649	2	23.824	0.719	0.489	0.012
	Obsessive‐compulsive (OC)	139.922	2	69.961	3.139	0.047	0.050
	Interpersonal sensitivity (IS)	38.661	2	19.330	1.038	0.357	0.017
	Depression (DE)	103.181	2	51.590	1.682	0.190	0.027
	Anxiety (AN)	27.272	2	13.636	0.687	0.505	0.011
	Hostility (HO)	104.947	2	52.473	5.381	0.006	0.082
	Phobic anxiety (PA)	2.772	2	1.386	0.209	0.811	0.003
	Paranoid ideation (PI)	9.600	2	4.800	0.669	0.514	0.011
	Psychoticism (PS)	31.609	2	15.804	1.082	0.342	0.018
Personality traits clustering × blood compensatory levels	Somatization (SO)	17.752	2	8.876	0.268	0.765	0.004
	Obsessive‐compulsive (OC)	70.644	2	35.322	1.585	0.209	0.026
	Interpersonal sensitivity (IS)	88.190	2	44.095	2.369	0.098	0.038
	Depression (DE)	163.756	2	81.878	2.670	0.073	0.043
	Anxiety (AN)	73.900	2	36.950	1.862	0.160	0.030
	Hostility (HO)	56.308	2	28.154	2.887	0.060	0.046
	Phobic anxiety (PA)	8.908	2	4.454	0.673	0.512	0.011
	Paranoid ideation (PI)	29.161	2	14.580	2.033	0.135	0.033
	Psychoticism (PS)	117.614	2	58.807	4.026	0.020	0.063

### 3.6 Results of Simple Effect Analysis

**FIGURE 5:**
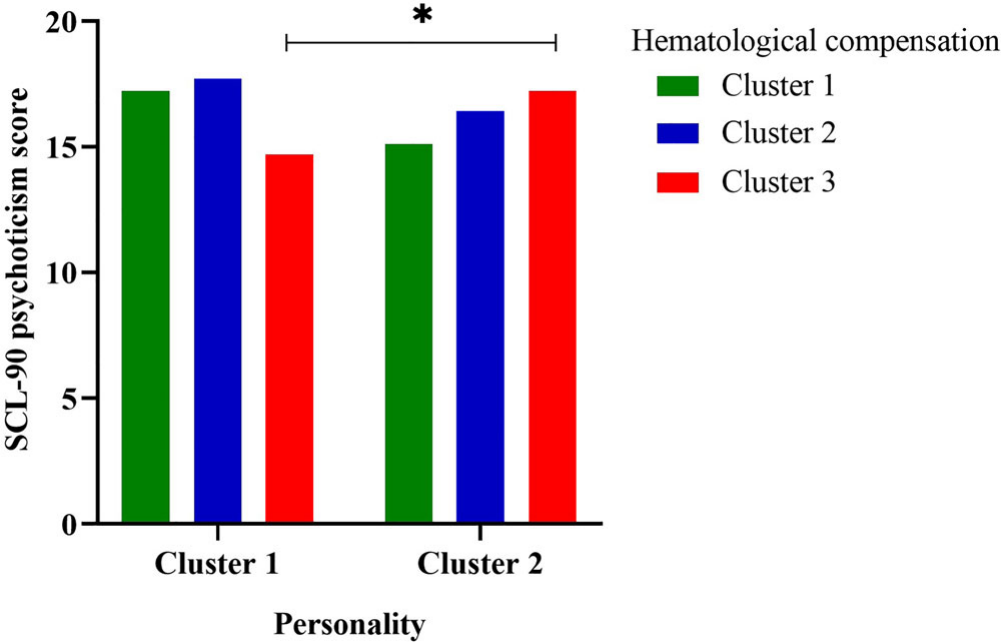
Simple effects of personality cluster within each hematologic compensation cluster on SCL‐90 psychoticism scores. *p < 0.05.

**FIGURE 6:**
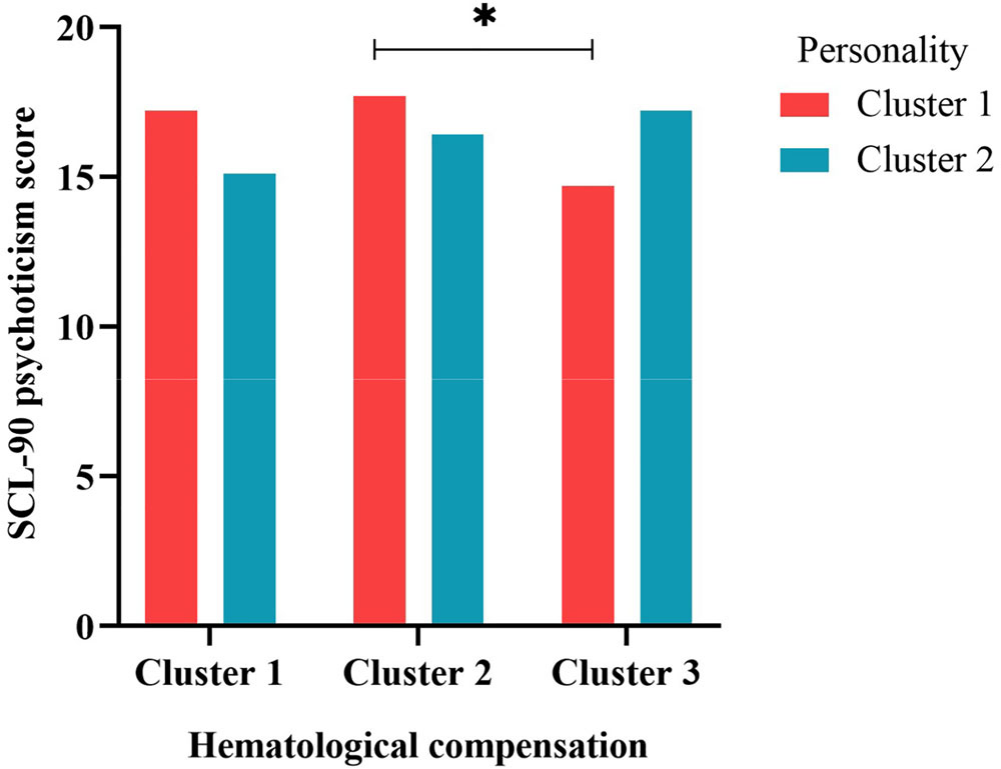
Simple effects of hematologic compensation cluster within each personality cluster on SCL‐90 psychoticism scores. *p < 0.05.

As shown in Tables 5, 6, 7 and Figures 5, 6, personality cluster differences in SCL‐90 psychoticism scores were significant only within the high hematologic compensation group. Specifically, within this group, the high‐neuroticism cluster reported lower SCL‐90 psychoticism scores than the low‐neuroticism cluster (estimate = −1.287, *t* = −2.203, *p* = 0.0295, 95% CI [−2.443, −0.131]).

**TABLE 5:** Estimated marginal means of SCL‐90 psychoticism scores by personality cluster and hematologic compensation cluster.

Blood cluster	Personality cluster	Estimated mean	SE	*df*	Lower CI	Upper CI	*t*‐ratio	*p*‐value
1	1	17.2	0.976	120	15.2	19.1	17.589	< 0.0001
2	1	17.7	0.702	120	16.3	19.1	25.258	< 0.0001
3	1	14.7	0.891	120	12.9	16.4	16.467	< 0.0001
1	2	15.1	1.09	120	12.9	17.2	13.827	< 0.0001
2	2	16.4	0.69	120	15.1	17.8	23.819	< 0.0001
3	2	17.2	0.756	120	15.7	18.7	22.811	< 0.0001

**TABLE 6:** Simple effects of personality cluster within each hematologic compensation cluster on SCL‐90 psychoticism scores.

Blood cluster	Personality cluster compared	Estimate	SE	*df*	Lower CI	Upper CI	*t*‐ratio	*p*‐value
1	1 vs. 2	1.039	0.732	120	−0.6215	2.6995	1.42	0.1582
2	1 vs. 2	0.645	0.492	120	−0.4711	1.7619	1.312	0.1921
3	1 vs. 2	−1.287	0.584	120	−2.443	−0.131	−2.203	0.0295

**TABLE 7:** Pairwise comparisons of hematologic compensation clusters within each personality cluster on SCL‐90 psychoticism scores.

Personality cluster	Contrast	Estimate	SE	*df*	Lower CI	Upper CI	*t*‐ratio	*p*‐value
1	Blood 1 vs. Blood 2	−0.563	1.2	120	−3.414	2.288	−0.468	0.8863
1	Blood 1 vs. Blood 3	2.495	1.32	120	−0.64	5.629	1.888	0.1464
1	Blood 2 vs. Blood 3	3.057	1.13	120	0.367	5.748	2.696	0.0216
2	Blood 1 vs. Blood 2	−1.35	1.29	120	−4.412	1.712	−1.046	0.5495
2	Blood 1 vs. Blood 3	−2.157	1.33	120	−5.305	0.992	−1.625	0.239
2	Blood 2 vs. Blood 3	−0.807	1.02	120	−3.234	1.621	−0.788	0.7109

Conversely, hematologic compensation differences were significant only within the high‐neuroticism cluster. In this personality profile, the high compensation group showed lower SCL‐90 psychoticism scores than the medium compensation group (estimate = 3.057, *t* = 2.696, *p* = 0.0216, 95% CI [0.367, 5.748]). No other simple effects reached statistical significance.

## 4 Discussion

This study examined the joint effects of personality trait clusters and hematologic compensation profiles on multiple dimensions of psychological symptoms in the context of chronic high‐altitude hypoxia. Overall, the interaction pattern was limited. Across most SCL‐90 dimensions, no significant personality cluster × hematologic compensation interactions were observed. A significant interaction emerged only for the SCL‐90 psychoticism dimension, suggesting a possible domain‐specific association between personality profile, hematologic compensation, and self‐reported psychoticism‐related symptom tendencies. Importantly, the SCL‐90 psychoticism dimension should not be interpreted as clinical psychosis or psychotic disorder. Rather, it reflects subclinical features such as interpersonal alienation, social withdrawal, unusual perceptual experiences, and thought‐related discomfort. Simple‐effect analyses indicated that within the high hematologic compensation group, individuals in the high‐neuroticism personality cluster showed lower SCL‐90 psychoticism scores than those in the low‐neuroticism cluster. However, given that this effect was confined to a single symptom domain, it should be interpreted cautiously as a preliminary and exploratory finding rather than as evidence for a general physiological buffering mechanism or a clinically meaningful psychosis‐related outcome.

One possible interpretation for the observed unexpected interaction pattern—wherein individuals high in neuroticism exhibited lower SCL‐90 psychoticism scores under high hematologic compensation—can be considered in relation to the physiological buffering model. This framework proposes that greater physiological adaptation or resource capacity may help reduce the psychological impact of stress exposure by limiting allostatic burden and supporting neural stability (McEwen 2004). Neurotic individuals are generally characterized by heightened emotional reactivity and perceptual sensitivity, which may increase their susceptibility to stress‐related psychological symptoms, including subclinical psychoticism‐related features such as interpersonal alienation, unusual perceptual experiences, and thought‐related discomfort (Lahey 2009). In the context of chronic high‐altitude hypoxia, higher hematologic compensation may reflect greater oxygen‐carrying capacity, which could be relevant to maintaining psychological functioning under hypoxic stress (Burtscher et al. 2022). In addition, neuroimaging studies have suggested that neural systems involved in emotion regulation, including prefrontal‐limbic networks, may be relevant to individual differences in emotional vulnerability and regulation capacity (Campbell‐Sills et al. 2011; Kebets et al. 2021). However, because the present study did not directly measure cerebral oxygenation, neural activity, or emotion‐regulation processes, this interpretation should be regarded as tentative. Thus, the counterintuitive finding may suggest that the association between neuroticism‐related personality profiles and self‐reported SCL‐90 psychoticism‐related tendencies varies across levels of hematologic compensation, rather than demonstrating a causal protective mechanism of physiological adaptation or a clinically defined psychosis‐related process.

An important point to consider is why the interaction between personality traits and hematologic compensation emerged specifically on the SCL‐90 psychoticism dimension, while no significant interaction effects were observed on other common symptom domains such as anxiety or DE. One possible explanation is that this SCL‐90 dimension captures self‐reported cognitive‐perceptual and interpersonal features, including unusual perceptual experiences, interpersonal alienation, social withdrawal, and thought‐related discomfort, which may differ from more affectively oriented symptom domains (Meehl 1962; Carter and Barch 2007). In contrast, symptoms such as anxiety and DE are often understood as more sustained affective states that are strongly related to long‐term trait vulnerabilities and accumulated environmental stressors, particularly neuroticism (Ormel et al. 2013). In the context of high‐altitude hypoxia, it is possible that SCL‐90 psychoticism‐related tendencies show different patterns of association with physiological adaptation than some other symptom domains. However, this possibility remains speculative because the present study did not directly assess perceptual processing, neurophysiological regulation, or cerebral oxygenation. Therefore, rather than suggesting a specific cognitive‐perceptual or clinical mechanism, the present finding should be interpreted more conservatively as a domain‐specific interaction pattern. Future studies using longitudinal designs, validated clinical or semi‐structured assessments, and neurobiological measures are needed to determine whether SCL‐90 psychoticism‐related tendencies are more sensitive to personality–physiology associations under chronic hypoxic exposure.

The simple effects analysis revealed a nonlinear interaction pattern. Specifically, within the high hematologic compensation group, individuals in the high‐neuroticism cluster exhibited lower SCL‐90 psychoticism scores than their low‐neuroticism counterparts. This pattern appears inconsistent with the general view that neuroticism is typically associated with elevated psychological risk (Lahey 2009). However, this finding should not be interpreted as evidence that neuroticism itself becomes protective under high hematologic compensation. Rather, it suggests that the association between neuroticism‐related personality profiles and self‐reported psychoticism‐related tendencies may differ depending on hematologic compensation level. Within the low and medium hematologic compensation groups, neuroticism showed no significant effect on SCL‐90 psychoticism scores, indicating that the personality‐related difference was not consistent across all physiological profiles. Similarly, among individuals in the low‐neuroticism cluster, SCL‐90 psychoticism scores did not significantly differ across hematologic compensation levels, suggesting that this interaction was mainly driven by the lower scores observed in the high‐neuroticism cluster under high hematologic compensation. Taken together, these results support a cautious interpretation of the interaction as a specific and conditional statistical pattern, rather than as evidence for a broad personality–environment–physiology mechanism or a clinical psychosis‐related effect.

The absence of significant main effects of personality cluster and hematologic compensation cluster on psychoticism suggests that the observed effect should not be interpreted as a straightforward association between either factor and psychoticism. Instead, the result indicates a conditional pattern that emerged only when personality and hematologic profiles were considered jointly. Specifically, the association between a given personality profile—such as high neuroticism—and psychological symptoms may not manifest uniformly across all physiological states but may vary depending on a moderator such as physiological adaptation. This logic is broadly consistent with the vulnerability × moderator model (Ingram and Luxton 2005), wherein latent psychological risks may become more or less apparent under specific biological or environmental conditions. When the moderator is at a certain level, such as high hematologic compensation, the association between a risk‐related trait profile and symptom expression may be attenuated or altered. Interaction effects of this kind are commonly considered in research on stress reactivity, trauma, and psychopathology (Monroe and Simons 1991). Nevertheless, because this interaction was limited to one outcome among multiple SCL‐90 dimensions, its theoretical significance should be considered tentative. Future studies with larger samples and preregistered hypotheses are needed to determine whether this pattern is replicable.

Beyond the psychoticism dimension, this study found main effects of personality and hematologic compensation clusters on specific SCL‐90 subscales, although no significant interaction effects emerged in these dimensions. Specifically, the high‐neuroticism cluster showed significantly higher scores only on the DE dimension, consistent with prior research linking neuroticism with elevated negative affect and depressive symptoms (Lahey 2009). Additionally, hematologic compensation showed significant main effects on the OC and HO dimensions, indicating that these symptom domains may vary across hematologic compensation levels (Testa et al. 2013). However, because these effects did not emerge as significant interactions, they should be interpreted as separate associations rather than evidence for personality–physiology coupling in these domains. Unlike the psychoticism dimension, DE, OC symptoms, and HO did not show evidence of a combined personality cluster × hematologic compensation pattern in the present sample. These findings further support a cautious, domain‐specific interpretation of the results and highlight the need for future research to examine whether distinct psychological symptom domains differ in their sensitivity to personality and physiological adaptation under chronic high‐altitude hypoxia.

Several limitations should be noted. First, although the study examined multiple SCL‐90 symptom dimensions, the personality cluster × hematologic compensation interaction was significant only for psychoticism. This limits the generalizability of the findings and suggests that the result should be considered domain‐specific and exploratory. Second, the cross‐sectional design precludes causal inference regarding whether hematologic compensation directly alters psychological symptom expression. Third, the sample size was modest for detecting interaction effects across multiple outcomes, and replication in larger high‐altitude cohorts is needed. Finally, the psychoticism dimension was measured using the SCL‐90, which reflects self‐reported subclinical psychoticism‐related tendencies rather than clinical psychosis or psychotic disorder. Therefore, the present findings should not be interpreted as evidence for clinical psychotic symptoms, psychotic disorder risk, or neurobiological mechanisms of psychosis.

It should also be noted that higher hematologic compensation is not necessarily beneficial in all contexts. The present participants were healthy long‐term high‐altitude residents, and the high hematologic compensation cluster represents relatively higher RBC, HGB, and HCT values within this non‐clinical cohort rather than chronic mountain sickness. Nevertheless, excessive erythrocytosis may become maladaptive when accompanied by ventilatory decline, severe hypoxemia, increased blood viscosity, or cardiopulmonary complications (Villafuerte et al. 2022). Because the present study did not assess ventilatory acclimatization, oxygen saturation, blood gas parameters, pulmonary hypertension, or chronic mountain sickness symptoms, we cannot determine whether similar hematologic patterns would have different implications in clinically vulnerable high‐altitude populations. Future studies should integrate hematologic, ventilatory, metabolic, and clinical indicators to distinguish adaptive compensation from maladaptive excessive erythrocytosis.

In summary, the present study provides preliminary evidence that personality profiles and hematologic compensation may jointly relate to psychoticism‐related symptoms in long‐term high‐altitude residents. However, the interaction effect was not observed across most psychological symptom dimensions, and therefore the findings should not be generalized to overall psychopathology. The results should be viewed as an initial observation that may guide future research on personality–physiology interactions under chronic hypoxic exposure. Longitudinal studies, larger samples, and additional physiological or neurobiological indicators are needed to clarify whether hematologic compensation plays a causal or moderating role in psychological adaptation to high altitude.

## 5 Conclusions

In the context of chronic high‐altitude hypoxia, this study used a dual‐clustering approach to examine whether personality profiles and hematologic compensation jointly relate to psychological symptoms. The main interaction was observed only for the SCL‐90 psychoticism dimension, suggesting a limited and domain‐specific pattern. Importantly, this dimension reflects self‐reported subclinical psychoticism‐related tendencies rather than clinical psychosis or psychotic disorder. Therefore, these findings should be interpreted cautiously and do not support a generalized buffering effect across psychological symptoms or a clinical interpretation of psychosis‐related outcomes. Future longitudinal and mechanistic studies are needed to determine whether hematologic compensation contributes to individual differences in psychological adaptation in high‐altitude environments.

## Author Contributions

**Zhibin Geng**: writing – review and editing, funding acquisition. **Wenjie Liu**: writing – review and editing. **Yuan Li**: conceptualization, methodology, formal analysis, investigation, resources, data curation, writing – original draft, writing – review and editing, visualization. **Hailin Ma**: investigation, resources, supervision, methodology, project administration, writing – review and editing. **Hao Li**: resources, investigation, supervision, project administration, writing – review and editing, funding acquisition. **Niannian Wang**: writing – review and editing, resources, data curation, funding acquisition.

## Funding

The research was supported by Central Government‐Guided Local Projects in the Xizang Autonomous Region (XZ202501YD0015), Science and Technology Plan Project of Xizang Autonomous Region (XZ202401ZY0106 and XZ202401ZY0011) and Plateau Tunnel Construction Project (cz02‐zx‐02).

## Ethics Statement

The study was approved by the Ethics Committee of Xizang University (ZDYXLL2024003) and conducted in accordance with institutional guidelines and regulations.

## Consent

Written informed consent was obtained from all participants prior to the experiment. All participants volunteered to participate in this study.

## Conflicts of Interest

The authors declare no conflicts of interest.

## Data Availability

The data and materials generated during and/or analyzed during the current study are available from the corresponding author on reasonable request.

## References

[brb371591-bib-0001] Bai, Y. , Y. Hu , Z. Zhou , X. Du , Y. Shi , and L. You . 2024. “Rise Above Prejudice Against Personality: Association With Personality and Interactive Collaboration in Team Creativity Performance.” Thinking Skills and Creativity 52: 101539. 10.1016/j.tsc.2024.101539.

[brb371591-bib-0002] Burtscher, J. , M. Niedermeier , K. Hüfner , et al. 2022. “The Interplay of Hypoxic and Mental Stress: Implications for Anxiety and Depressive Disorders.” Neuroscience & Biobehavioral Reviews 138: 104718. 10.1016/j.neubiorev.2022.104718. 35661753

[brb371591-bib-0003] Campbell‐Sills, L. , A. N. Simmons , K. L. Lovero , A. A. Rochlin , M. P. Paulus , and M. B. Stein . 2011. “Functioning of Neural Systems Supporting Emotion Regulation in Anxiety‐Prone Individuals.” Neuroimage 54, no. 1: 689–696. 10.1016/j.neuroimage.2010.07.041. 20673804 PMC2962684

[brb371591-bib-0004] Carter, C. S. , and D. M. Barch . 2007. “Cognitive Neuroscience‐Based Approaches to Measuring and Improving Treatment Effects on Cognition in Schizophrenia: The CNTRICS Initiative.” Schizophrenia Bulletin 33, no. 5: 1131–1137. 10.1093/schbul/sbm081. 17630405 PMC2632368

[brb371591-bib-0005] Dang, W. , Y. Xu , J. Ji , et al. 2021. “Study of the SCL‐90 Scale and Changes in the Chinese Norms.” Frontiers in Psychiatry 11: 524395. 10.3389/fpsyt.2020.524395. 33584353 PMC7873442

[brb371591-bib-0006] Du, L. , H. Dong , C. Miao , F. Jia , and L. Shan . 2022. “Analysis of Scores of Symptom Checklist 90 (SCL‐90) Questionnaire of 182 Parents of Children With Spinal Muscular Atrophy: A Cross‐Sectional Study.” Translational Pediatrics 11, no. 11: 1776–1786. 10.21037/tp-22-464. 36506780 PMC9732609

[brb371591-bib-0007] Gong, W. , C. Fu , S. Guo , X. Chen , and C. Zheng . 2025. “Effects of Chronic Hypoxic Exposure on the Attention Network of Indigenous Plateau Primary School Students.” Preprint, SSRN June 9. https://ssrn.com/abstract=5279388.

[brb371591-bib-0008] Heinrich, E. C. , M. A. Djokic , D. Gilbertson , et al. 2019. “Cognitive Function and Mood at High Altitude Following Acclimatization and use of Supplemental Oxygen and Adaptive Servoventilation Sleep Treatments.” PLoS ONE 14, no. 6: e0217089. 10.1371/journal.pone.0217089. 31188839 PMC6561544

[brb371591-bib-0009] Hösl, B. , M. Niedermeier , J. Burtscher , and M. Kopp . 2025. “Psychological Effects of Mountainous Environments Over the Life Span and Potential Implications for Healthy Ageing: A Narrative Review.” Gerontology 71, no. 7: 546–554. 10.1159/000546367. 40418904

[brb371591-bib-0010] Ingram, R. E. , and D. D. Luxton . 2005. “Vulnerability‐Stress Models.” In Development of Psychopathology: A Vulnerability‐Stress Perspective, edited by B. L. Hankin and J. R. Z. Abela , 32–46. SAGE Publications, Inc. 10.4135/9781452231655.n2.

[brb371591-bib-0011] Kebets, V. , P. Favre , J. Houenou , et al. 2021. “Fronto‐Limbic Neural Variability as a Transdiagnostic Correlate of Emotion Dysregulation.” Translational Psychiatry 11, no. 1: 545. 10.1038/s41398-021-01666-3. 34675186 PMC8530999

[brb371591-bib-0012] Lahey, B. B. 2009. “Public Health Significance of Neuroticism.” American Psychologist 64, no. 4: 241–256. 10.1037/a0015309. 19449983 PMC2792076

[brb371591-bib-0013] Li, F. , X. Zhang , A. Ye , et al. 2025. “The Effects and Mechanisms of Continuous 7‐Day Hypobaric Hypoxia Exposure on Sleep Architecture in Rats.” International Journal of Molecular Sciences 26, no. 11: 4998. 10.3390/ijms26114998. 40507808 PMC12155171

[brb371591-bib-0014] Li, M. , B. Zhang , and Y. Mou . 2024. “Though Forced, Still Valid: Examining the Psychometric Performance of Forced‐Choice Measurement of Personality in Children and Adolescents.” Assessment 32, no. 4: 521–543. 10.1177/10731911241255841. 38867477

[brb371591-bib-0015] Li, Y. , N. Wang , F. Hu , H. Ma , and H. Li . 2025. “Adaptive Hematological Profiles and Brain Structure Buffer Depression in High‐Altitude Healthy Adults.” Biological Psychology 202: 109155. 10.1016/j.biopsycho.2025.109155. 41173169

[brb371591-bib-0016] Liu, Y. , C. J. Hopwood , A. L. Pincus , et al. 2024. “Interpersonal Problem Profiles of Personality and Psychopathology Constructs in Chinese Undergraduates and Offenders.” Assessment 32, no. 2: 253–268. 10.1177/10731911241241495. 38606887

[brb371591-bib-0017] McEwen, B. S. 2004. “Protection and Damage from Acute and Chronic Stress: Allostasis and Allostatic Overload and Relevance to the Pathophysiology of Psychiatric Disorders.” Annals of the New York Academy of Sciences 1032, no. 1: 1–7. 10.1196/annals.1314.001. 15677391

[brb371591-bib-0018] Meehl, P. E. 1962. “Schizotaxia, Schizotypy, Schizophrenia.” American Psychologist 17, no. 12: 827–838. 10.1037/h0041029.

[brb371591-bib-0019] Monroe, S. M. , and A. D. Simons . 1991. “Diathesis‐Stress Theories in the Context of Life Stress Research: Implications for the Depressive Disorders.” Psychological Bulletin 110, no. 3: 406–425. 10.1037/0033-2909.110.3.406. 1758917

[brb371591-bib-0020] Ochsner, K. N. , and J. J. Gross . 2008. “Cognitive Emotion Regulation.” Current Directions in Psychological Science 17, no. 2: 153–158. 10.1111/j.1467-8721.2008.00566.x. 25425765 PMC4241349

[brb371591-bib-0021] Ormel, J. , B. F. Jeronimus , R. Kotov , et al. 2013. “Neuroticism and Common Mental Disorders: Meaning and Utility of a Complex Relationship.” Clinical Psychology Review 33, no. 5: 686–697. 10.1016/j.cpr.2013.04.003. 23702592 PMC4382368

[brb371591-bib-0022] Stalmans, M. , D. Tominec , W. Lauriks , et al. 2025. “Ketone Ester Ingestion Impairs Exercise Performance without Impacting Cognitive Function or Circulating EPO During Acute Hypoxic Exposure.” Journal of Applied Physiology 138, no. 6: 1309–1320. 10.1152/japplphysiol.00097.2025. 40315254

[brb371591-bib-0023] Testa, A. , R. Giannuzzi , S. Daini , L. Bernardini , L. Petrongolo , and N. G. Silveri . 2013. “Psychiatric Emergencies (part III): Psychiatric Symptoms Resulting From Organic Diseases.” European Review for Medical and Pharmacological Sciences 17, no. S3: 86–99. https://pubmed.ncbi.nlm.nih.gov/23436670. 23436670

[brb371591-bib-0024] Villafuerte, F. C. , T. S. Simonson , D. Bermudez , and F. León‐Velarde . 2022. “High‐Altitude Erythrocytosis: Mechanisms of Adaptive and Maladaptive Responses.” Physiology 37, no. 4: 175–186. 10.1152/physiol.00029.2021. PMC9191173 35001654

[brb371591-bib-0025] Widiger, T. A. , and J. R. Oltmanns . 2017. “Neuroticism is a Fundamental Domain of Personality With Enormous Public Health Implications.” World Psychiatry 16, no. 2: 144–145. 10.1002/wps.20411. 28498583 PMC5428182

[brb371591-bib-0026] Xu, H. , L. Peng , Z. Wang , and X. Liu . 2022. “Effects of Psychological Capital and Social Support Availability on Anxiety and Depression Among Chinese Emergency Physicians: Testing Moderated Mediation Model.” Frontiers in Psychology 13: 991239. 10.3389/fpsyg.2022.991239. 36571060 PMC9768176

[brb371591-bib-0027] Xu, Y. , Q. Hua , W. Chen , et al. 2025. “Human Adaptation to High Altitude: Acclimatization and Reversibility of Haemodynamics.” National Science Review 12, no. 7: nwaf203. 10.1093/nsr/nwaf203. 40585565 PMC12202200

[brb371591-bib-0028] Zhang, B. , Y. M. Li , J. Li , et al. 2021. “The Big Five Inventory‐2 in China: A Comprehensive Psychometric Evaluation in Four Diverse Samples.” Assessment 29, no. 6: 1262–1284. 10.1177/10731911211008245. 33884926

